# Canine Rabies in the Brazil-Bolivia Border Region from 2006 to 2014

**DOI:** 10.5334/aogh.2334

**Published:** 2019-03-04

**Authors:** Juliana Arena Galhardo, Carla Silva de Azevedo, Bárbara Ribeiro Remonti, Vitória Machado Neres Gonçalves, Natalia Trindade Azevedo Marques, Lilian Oliveira Borges, Danielle Ahad das Neves

**Affiliations:** 1 Federal University of Mato Grosso do Sul, Veterinary Medicine and Animal Husbandry Faculty, Campo Grande, Mato Grosso do Sul, BR; 2 State Agency of Animal and Plant Health Protection of Mato Grosso do Sul, Animal Diseases Diagnostic Laboratory, Campo Grande, Mato Grosso do Sul, BR

## Abstract

**Background:**

After 2006 the cross-border region between the state of Mato Grosso do Sul (Brazil) and the Germán Busch Province (Bolivia) became risk areas for canine rabies antigenic variant 1, previously unknown in the Brazilian territory.

**Objectives:**

To perform a descriptive analysis of canine rabies from 2006 to 2014, analyzing the database of the official rabies diagnostic laboratory of the State Agency of Animal and Plant Health Protection of Mato Grosso do Sul.

**Methods:**

A descriptive analysis of canine rabies from 2006 to 2014 was performed using the database of the official rabies diagnostic laboratory of the State Agency of Animal and Plant Health Protection of Mato Grosso do Sul. Location, time and residence status of the animals were analyzed. Monthly frequencies were calculated as the ratio of the number of positive samples to the total of sent samples and were then statistically compared.

**Findings:**

In the period, 539 samples of nervous system from dogs and cats were sent for rabies diagnosis, of which 37 (6.9%; CI95% 5.0–9.3) canine and no positive feline samples were found positive. Twenty-four (64.9%, CI95% 48.8–78.2) positive samples were from Bolivia and 13 (31.1%, CI95% 21.8–51.2) from Brazil. Most positive animals were owned. The years 2008 and 2009 showed the highest occurrence of canine rabies, with 18 cases recorded in 2008 and 6 in 2009 (17 in Bolivia and 7 in Brazil). Annual samples sent in Brazil presented a decreasing trend (R^2^ = 0.53) and, over the months, a higher concentration of samples was observed between May and August (R^2^ = 0.69). No annual or monthly trends were observed for Bolivian samples (R^2^ < 0.003).

**Conclusions:**

AgV1 canine rabies due to antigenic variant 1 is still considered an endemic disease in the Brazil-Bolivia border region, requiring an international One Health Approach to mitigate canine rabies in Latin America.

## Introduction

Rabies is a transmissible viral encephalomyelitis, of a zoonotic nature caused by *Rabies lyssavirus* (RABV), belonging to the Order *Mononegavirales*, Family *Rhabdoviridae* and Genus *Lyssavirus* [1]. In the Americas, RABV is classified in 12 antigenic variants (AgV) according to the Centers for Diseases Control and Prevention (CDC/USA) and the domestic dog is the reservoir of AgV1 and AgV2. AgV1 circulates in Latin America and was introduced in Brazil in 2006 through the Brazil-Bolivia border; AgV2 circulates mainly in Brazil, Argentina, Bolivia and Paraguay [2,3,4,5].

Since the beginning of the Pan American Health Organization (PAHO) Rabies Elimination Program, Latin America and the Caribbean have experienced a 95% reduction in the number of human rabies cases, from 355 cases in 1983 to less than 10 in 2012. There was a 98% reduction in canine cases – from 25,000 in 1980 to 400 in 2010 [6,7,8,9], but despite efforts to control rabies, outbreaks still persist in urban areas of Latin America, denoting failures in the surveillance system.

According to the PAHO, Bolivia and Haiti have the worst conditions in urban rabies control compared to all Latin America countries. In Haiti, the situation remains due to environmental disasters that have recently devastated the country, and in Bolivia for the lack of financial resources, poor policies for dog vaccination, a large semi-confined owned or unowned dog population and disorganized urbanization. The department of Santa Cruz and the city of Santa Cruz de la Sierra, Bolivia’s most populous municipality, are also considered endemic areas for rabies transmitted by dogs and the underreporting of canine cases is a persistent problem in the region [10,11,12].

Since the 1950s several Brazilian municipalities have started activities to control human rabies, which included dog vaccination, blocking of outbreaks and capture-culling of unowned dogs. After 1973, with the implementation of the National Human Rabies Prophylaxis Program, vaccination campaigns were intensified, canine rabies due to AgV2 was eliminated in most of the country and was less implicated in outbreaks. The reduction of canine cases brought a reduction in the number of human cases, but more recently a change was observed in the epidemiological profile of rabies transmission in Brazil, with the detection of human cases transmitted by bats and the greater participation of other wild species, as well as and the emergence of dog-transmitted AgV1 in the Brazil-Bolivia border from 2006 on, which demands new surveillance strategies [13,14,15].

Considering the need to understand the epidemiological aspects of canine rabies transmission in the Brazil-Bolivia border, the aim of this work was to perform a descriptive analysis of canine rabies from 2006 to 2014, analyzing the database of the official rabies diagnostic laboratory of the State Agency of Animal and Plant Health Protection of Mato Grosso do Sul.

## Methods

The study area was the dry border region between Brazil and Bolivia, including the cities of Corumbá and Ladário (Mato Grosso do Sul State, Brazil) and Puerto Quijarro and Puerto Suárez (Germán Busch Province, Bolivia). Puerto Quijarro is located only 4.5 km away from Corumbá and the total distance between the urban areas of Puerto Suárez (West) and Ladário (East) is 21 km. There are no natural barriers that prevent the flow of people or animals in the region. The urban areas of Corumbá and Ladário are contiguous and are three kilometers away, as well as Puerto Quijarro and Puerto Suárez, which are 10 km away.

The urban rabies active surveillance service of Corumbá and Ladário is carried out by agents of the Municipal Health Secretariats of both municipalities and basically consists of capture and culling of dogs and cats along with the collection of central nervous system (CNS) material at the Zoonoses Surveillance Unit (ZSU) of Corumbá. The ZSU sends the CNS samples to the official rabies diagnostic laboratory of the State Agency of Animal and Plant Health Protection of Mato Grosso do Sul (IAGRO), which performs the direct immunofluorescence and biological test techniques [16]. Rabies virus antigenic variant confirmation was performed by Pasteur Institute in São Paulo. Bolivian samples were mainly from symptomatic dogs and cats voluntarily delivered to ZSU by owners or health agents. Brazil only carries out actions of urban rabies surveillance in the Bolivian territory under bilateral cooperation agreements.

In order to perform a descriptive analysis of urban rabies in the Brazil-Bolivia border region between 2006 and 2014, the IAGRO database was analyzed evaluating the information about samples sent by ZSU in the period. The records were organized into spreadsheets using Apache software OpenOfficeCalc 4.1.1 (openoffice.org). The variables species (dog or cat), location (municipality), residence status of the animal (owned or unowned) and time (year and month of sample submission) were analyzed. The monthly frequencies were calculated as the proportion of the number of positive samples in relation to the total of sent samples. Statistical analyzes were performed using Bioestat 5.3 and Past 3.21 software. The variables gender, age, breed and vaccination status were not analyzed due to the absence of such information in the original database.

## Results

In nine years (2006 to 2014) 539 CNS samples were sent for rabies diagnosis, 508 from dogs, 27 from cats and 4 from unspecified species. Of the 539 samples, 37 (6.9%; 95% CI 5.0–9.3) were positive for rabies, all AgV1 canine samples. Of these, 24 (64.9%; 95% CI 48.8–78.2) came from Bolivia and 13 (31.1%; 95% CI 21.8–51.2) from Brazil. As of 2010, the number of samples sent from Corumbá and Ladário presented a decreasing trend (R^2^ = 0.53) and over the months, a higher concentration of samples was observed between May and August (R^2^ = 0.69). No annual or monthly trends were observed for Bolivian samples (R^2^ < 0.003). Annual positivity rate is shown in Table 1 whereas monthly positivity rate is shown in Table 2.

**Table 1:** Dog and cat central nervous system samples from the Brazil-Bolivia border region sent for rabies diagnosis to the State Agency for Animal and Plant Health Protection of Mato Grosso do Sul by the Zoonoses Surveillance Unit of Corumbá, from 2006 to 2014.

Country	Sample	Year								

		2006	2007	2008	2009	2010	2011	2012	2013	2014

**Brazil**	Sent	61	65	96	73	49	44	40	52	15
	Positive	1	4	6	1	0	0	1	0	0
	Positivity rate(%)	1.6	6.2	6.3	1.4	0	0	2.5	0	0

**Bolivia**	Sent	1	NS	17	17	4	1	2	NS	2
	Positive	1	–	12	5	3	0	1	–	2
	Positivity rate(%)	100	–	70.6	29.4	75	0	50	–	100

*NS = not sent.

**Table 2:** Dog and cat central nervous system samples from the Brazil-Bolivia border region sent for rabies diagnosis to the State Agency for Animal and Plant Health Protection of Mato Grosso do Sul by the Zoonoses Surveillance Unit of Corumbá, arranged by month over the period of 2006 to 2014.

Country	Sample	Month											

		Jan	Feb	Mar	Apr	May	Jun	July	Aug	Sep	Oct	Nov	Dec

**Brazil**	Sent	23	61	52	82	120	73	114	122	74	70	41	51
	Positive	1	1	1	1	2	1	0	4	1	1	0	0
	Positivity rate(%)	4.3	1.6	1.9	1.2	1.7	1.4	0	3.3	1.4	1.4	0	0

**Bolivia**	Sent	2	6	14	NS	6	2	3	6	NS	6	1	NS
	Positive	1	4	3	–	4	2	1	4	–	4	1	–
	Positivity rate(%)	50	66.7	21.4	–	66.7	100	33.3	66.7	–	66.7	100	–

*NS = not sent.

In this period, 456 samples were sent by the municipality of Corumbá, of which 11 were positive samples (2.4%; 95% CI 1.28–4.15). The municipality of Ladário sent 39 samples, with two positive results (5.1%; 95% CI 0.87–15.93). Regarding Bolivia, there were 31 samples from Puerto Suárez with 15 positive findings (48.4%; 95% CI 31.1–65.7) and 13 samples from Puerto Quijarro with 9 positive results (69.2%; 95% CI 41.3–89.4).

Concerning the residence status, 78.3% (422/539; 95% CI 74.6–81.6) of the animals were owned and of these, 35 were positive for rabies (8.3%; 95% CI 6.0–11.3). The unowned animals represented 21.7% (117/539; 95% CI 18.4–25.4) of the samples, of which 115 (98.3%) were from Brazil (107 animals from Corumbá and 8 from Ladário) and 2 from Puerto Quijarro. Of the unowned animals, only two dogs were positive for rabies, both from Corumbá (1.7%; 95% CI 0.5–6.0).

## Discussion

In 2006, the municipality of Corumbá recorded the first case of AgV1 canine rabies, not detected in Brazil until then [14]. In this study, the peak of occurrence of canine rabies in the border region was observed in 2008, with 6 cases occurring in Brazil and 12 in Bolivia. Fonseca [17] reports on the absence of specific legislation in Bolivia relating to the capture and destination of unowned dogs, in addition to the border population habitually keeping their dogs loose by the roads, which characterizes the semi-confined owned dogs.

Brandão [18] also highlighted the high number of unowned or semi-confined owned dogs, the absence of mechanisms to control the entry of animals at the border and the difficulty of keeping aggressive animals under observation as determining factors in the continuity of AgV1 transmission in the Brazil-Bolivia border region. The factors mentioned above are also associated with the increase in cases registered in 2008. This set of failures in the surveillance and control of urban rabies in the Brazil-Bolivia border region possibly contributed to the increase in the number of canine cases between 2008 and 2009.

The existence of semi-confined owned dogs could not be identified in this study since this information was not available. However, it is hypothesized that a portion of owned animals sent for rabies diagnosis from 2006 to 2014 may fit this criterion, as about 8% of the positive results came out of nearly 80% of the samples collected from owned dogs, whereas only 2 animals from 117 unowned were positive.

According to the recommendations of the National Rabies Control and Prophylaxis Program and the agreements among health authorities in Brazil, one of the actions of urban rabies surveillance is the annual analysis of at least 0.2% of the estimated canine population to the diagnosis of canine rabies [15]. It is estimated that 45 to 50 annual samples would be necessary for urban rabies surveillance in the two municipalities of Corumbá and Ladário. The decreasing trend in the number of samples sent for rabies diagnosis demonstrates an important failure in Brazilian active surveillance and possibly contributed to the continuity of the RABV transmission in the Brazil-Bolivia border region leading to the epizootic of 2015 [19]. Bolivian samples were sent to diagnosis in Brazil were mainly due to passive surveillance and the difficult access to laboratories in Bolivia; therefore, it was not possible to establish any trends for canine rabies positivity for these samples.

Brazil adopted a house-to-house biannual vaccination of dogs and cats in 2007 [14,18], a practice that collaborated with the control of urban rabies transmission and, despite the decreasing trend in sending samples for rabies diagnosis, the number of positive dogs in Brazil was also declining.

Given this situation, Wada et al. [14] emphasize the need for permanent and integrated surveillance and cooperation between border countries focusing on the different components of the rabies transmission chain. In other words, to successfully control urban rabies, it is necessary to associate vaccination coverage above 80% with dog population control, responsible pet ownership, health education and post-exposure prophylaxis for injured persons [20]. Some countries that have succeeded in controlling canine rabies, such as the United States, Canada, and Japan, have adopted policies like mandatory registration of animals, border control, systematic vaccination, import control of animals and quarantine and emergency action plans which are based on scientific evidence and are periodically revised [21,22].

The main contribution of this research was to highlight that the Brazil-Bolivia border region remains an enzootic area for canine rabies and that failures in active and passive surveillance provided the entry and permanence of AgV1 rabies in the Brazilian territory. Thus, urgent and integrated prevention and control measures along with policies of responsible pet ownership and health education, are required not only among the competent Municipal, State and Federal agencies but also among the population, to avoid the spread of the antigenic variant 1 to other regions of Brazil.

## Author Information

All authors had access to the data and played a role in writing the manuscript.
